# The Impact of Cocoa Flavanols in Modulating Resting Cerebral Blood Flow During Prolonged Sitting in Healthy Young and Older Adults

**DOI:** 10.3390/nu17132099

**Published:** 2025-06-24

**Authors:** Alessio Daniele, Samuel J. E. Lucas, Catarina Rendeiro

**Affiliations:** 1 School of Sport, Exercise and Rehabilitation Sciences, University of Birmingham, Birmingham B15 2TT, UK; axd905@student.bham.ac.uk (A.D.); s.j.e.lucas@bham.ac.uk (S.J.E.L.); 2 Centre for Human Brain Health, University of Birmingham, Birmingham B15 2TT, UK

**Keywords:** cocoa flavanols, sitting, brain, blood flow, aging

## Abstract

**Background:** Sitting is highly prevalent among young and older adults and can transiently reduce cerebral blood flow. Dietary flavanols confer benefits to the peripheral vasculature and may be effective at counteracting the impact of sitting in the cerebrovasculature. The aim of this study was to investigate whether the acute ingestion of flavanols prior to sitting improves common carotid artery (CCA) blood flow/shear rate (BF/SR) in young and older individuals. **Methods:** Two acute randomized, double-blinded, cross-over, placebo-controlled studies were conducted in 40 healthy young males (high-fit: 22.2 ± 2.9 yr., low-fit: 23.2 ± 4.1 yr., *N* = 20 per group) and 20 healthy older adults (72.4 ± 5.0 yr.). Participants consumed either a high- (695 mg) or low-flavanol (5.6 mg) cocoa beverage just before a 2 h sitting bout. Resting CCA retrograde/anterograde BF and SR, as well as arterial diameter, were assessed before and after the intervention. **Results:** Sitting reduced anterograde BF and/or SR in young and older individuals (*p* < 0.001) but only resulted in increases in retrograde BF (*p* = 0.021) and SR (*p* = 0.022) in the older group. Flavanols did not affect anterograde BF/SR in either group (*p* > 0.05) but mitigated (non-significant interaction: *p* = 0.053) sitting-induced increases in retrograde BF/SR in older individuals, with retrograde BF (*p* = 0.028) and SR (*p* = 0.033) increasing significantly only after intake of the low-flavanol beverage. No changes in arterial diameter were detected. **Conclusions:** This suggests that flavanols may have the potential to attenuate the detrimental sitting-induced increases in retrograde BF and SR in older adults, although larger well-powered studies are required to confirm this.

## 1. Introduction

The worldwide prevalence of cognitive impairments is currently estimated to be ~20% [1,2]. Approximately one-third of people suffering from cognitive impairments develop dementia within 5 years [3,4]. According to the World Health Organization [5], dementia is one of the main causes of disability among older populations, and it is projected to triple by 2050 [6]. Cognitive impairments in older adults, including dementia, have been consistently linked to reductions in cerebral blood flow (BF) or hypoperfusion [7]. Critically, lifestyle factors, such as physical inactivity and sedentary behavior [8,9,10], as well as unhealthy diets [11,12], have been shown to contribute to an accelerated decline in brain function.

Sedentary behavior is highly prevalent [13,14], with both young and older adults estimated to sit for more than 7 and 9 h/day, respectively [15,16]. Increased sitting time is associated with an increased risk for several chronic conditions, including cardiovascular disease (CVD), type 2 diabetes, obesity, and hypertension [17,18,19,20,21], which are all known risk factors for dementia and cognitive decline. Indeed, recent systematic reviews report positive associations between increased sedentary behavior and cognitive decline [22] and the incidence of dementia in aging [10]. Sedentary behavior is also associated with higher arterial stiffness in the common carotid artery (CCA) [23,24] and an increased prevalence of plaque in the carotid arteries [25], both of which are linked to reductions in cerebral perfusion and impaired cognitive function [26,27,28,29].

Critically, modulation of the brain vasculature can occur within a few hours of sitting uninterruptedly. For example, it has been shown that 1 to 8 h of sitting reduces blood velocity in the middle cerebral artery (MCA) in young [30] and overweight/obese older adults [31]. Decreased BF in the upstream internal carotid artery (ICA) and CCA BF in young healthy adults has also been reported, with no significant changes in the vertebral artery (VA) [32,33,34]. Studies in the CCA have been less consistent, with some studies describing no changes in BF alongside increases/no changes in carotid shear rate (SR) and arterial constriction [35,36,37]. Acute sitting may also temporarily impair cerebral autoregulation [38] and increase cerebrovascular resistance [39], which can have long-term deleterious effects on the brain’s vascular function and structure, resulting in global cognitive decline [40,41,42].

Lifestyle strategies, such as physical activity and nutrition, may be used to more precisely counteract impairments in the cerebral vasculature during sitting and consequently alleviate the long-term impact of inactivity on brain function. For example, walking and stair climbing breaks were efficacious in preventing CCA constriction during sitting [37], and a moderate-intensity walk prior to sitting can mitigate declines in MCA blood velocity [30,31]. Additionally, nutritional strategies may also be used to protect cerebral BF during sitting, particularly in older populations, who are more likely to sit for longer periods with no regular breaks [43]. However, few dietary approaches aimed at protecting the brain have been investigated in the context of sitting. One recent randomized controlled trial reported that the glycaemic index of a meal had no impact on cerebral BF and cognitive function during sitting [32].

Flavanols are a class of plant-derived flavonoids present in foods such as unprocessed cocoa, tea, grapes, apples, and berries [44]. Observational studies have shown that diets rich in flavonoids can protect against cognitive decline in aging [45,46,47]. Interestingly, a recent randomized controlled trial in 3562 older adults showed that a 3-year intervention with cocoa flavanols improved memory in individuals with poorer diets after 1 year, with improvements sustained over the 3-year follow-up period [48]. The effects of dietary flavanols on the peripheral conduit arteries are well-established, with improvements in nitric oxide (NO)-dependent flow-mediated dilation (FMD) increasing within 1–4 h of intake [49,50,51]. Interestingly, randomized controlled trials in young and older adults suggest that dietary flavanols can modulate the brain vasculature by increasing cerebral oxygenation levels, cerebral tissue perfusion, and blood velocity within a few hours of intake [52,53,54,55]. There is some evidence to suggest that, similar to the effects in the periphery, modulation of the cerebrovasculature may also be NO-mediated [54]. As such, flavanol-rich foods may be effective at alleviating transient sitting-induced declines in cerebral BF. Importantly, these strategies may be more critical in older individuals or those who are more sedentary and less fit, compared to highly fit individuals who typically have better peripheral and cerebral vascular function [56,57,58,59,60,61,62]. Indeed, aging is accompanied by a deterioration in vascular health, as indicated by greater arterial stiffness, diminished endothelial function, and increased arterial calcification [63,64], potentially heightening the susceptibility of older adults to the detrimental impact of prolonged sitting. Furthermore, it is widely recognized that athletes have enhanced cardiovascular health and a lower risk of CVD than the general population [57,61]. It is conceivable that higher levels of cardiorespiratory fitness confer vascular protection against the adverse effects of prolonged sitting, whilst less fit individuals may be more susceptible. Flavanol interventions may have differential benefits depending on aging and cardiorespiratory fitness.

Therefore, the aim of this study was to examine whether the acute consumption of dietary flavanols prior to a 2 h bout of uninterrupted sitting was effective at improving resting CCA BF and SR in healthy high- and low-fit young adults and healthy older adults. Anterograde and retrograde BF and SR will also be assessed separately, given that these parameters describe different functional aspects of the vasculature, which are masked when looking at variations in mean BF [65,66,67,68]. It was hypothesized that flavanols would be efficacious in modulating the cerebrovascular response during sitting in older adults and low-fit young adults, but not in high-fit young individuals.

## 2. Materials and Methods

### 2.1. Ethical Approval

The studies were conducted in accordance with the Declaration of Helsinki and approved by the University of Birmingham Science, Technology, Engineering, and Mathematics ethics committee (ERN_19-0851; ERN_19-0851B). Informed written consent was obtained from all participants before enrolment in the studies.

### 2.2. Participants

Forty young healthy male adults (aged 18–45 years old), either high fit (VO_2_peak ≥ 49 mL∙kg^−1^∙min^−1^, *N* = 20) or low fit (VO_2_peak ≤ 41 mL∙kg^−1^∙min^−1^, *N* = 20), and 20 healthy older adults (aged ≥ 65 years old, 13 females and 7 males) were recruited from the University of Birmingham (Birmingham, UK), the surrounding community, and “the Birmingham 1000 Elders group” to take part in two randomized, counterbalanced, within-subject controlled trials. Prior to participation in this study, all participants provided a signed informed consent form and completed a general health and lifestyle questionnaire. Individuals were excluded if they were smokers, on weight-reducing diets, had recently undergone prolonged bed-rest periods, or had a history or symptoms of cardiovascular, renal, pulmonary, metabolic, or neurologic disease, hypertension (blood pressure higher than 140/90 mm Hg based on recent guidelines [69]), diabetes mellitus, anemia, asthma, immune conditions, or high cholesterol. For the young group, individuals were excluded if they were using prescribed/over-the-counter medications or had a VO_2_peak between 41 and 49 mL∙kg^−1^∙min^−1^; young premenopausal females were not included in this study to minimize biological variation caused by fluctuations in female sex hormones across the menstrual cycle [70,71], as this was the first proof-of-principle study investigating a three-way interaction between levels of physical fitness, sitting, and dietary intervention. For the older group, individuals were excluded if they were taking anticoagulants. A fitness assessment using the VO_2_peak test in the older group was not possible for this study due to ethical considerations regarding participant safety, but 7-day average activity levels were monitored for all groups. The rationale behind these differentiated criteria lies in the understanding of health profiles across age groups. For young adults, the absence of prescribed/over-the-counter medications is a marker of good health; conversely, for older adults, the presence of prescribed/over-the-counter medications does not necessarily indicate poor health but rather reflects a normal aspect of aging, where such medications are frequently utilized for minor disorders or preventive care. Anticoagulant use was the only specified exclusion for older participants, as these medications can adversely affect vascular function [72] and introduce variables that may affect the study outcomes.

### 2.3. Study Design

**Figure 1:**
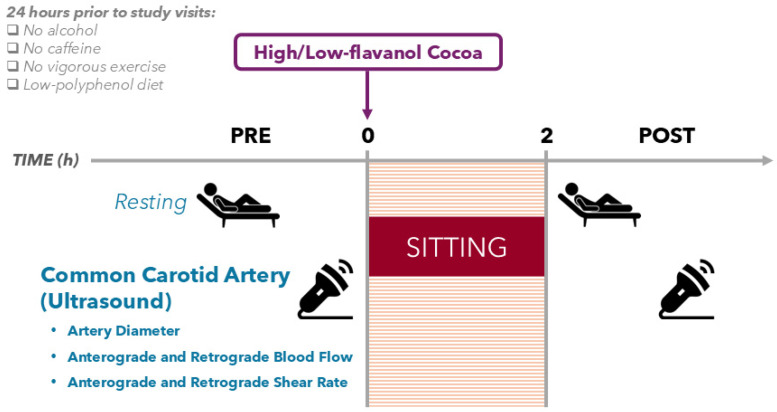
Experimental study design overview, featuring an ultrasound recording of the common carotid artery before and after a 2 h sitting trial.

Data presented within this study are secondary outcome measures combined from two randomized, double-blinded, counterbalanced, placebo-controlled, cross-over intervention studies [73,74]. The first was conducted in young high- and low-fit healthy adults (*N* = 40), and the second in older healthy adults (*N* = 20). Both studies consisted of 2 experimental visits separated by at least 7 days and followed the exact same protocol (Figure 1). Secondary outcome measures of CCA BF and SR (anterograde and retrograde) and arterial diameter are reported.

Briefly, before enrolment in this study, young adults completed a preliminary session in which their maximal aerobic capacity was assessed to determine eligibility. Participants’ anthropometric characteristics (height and weight) were measured before the first experimental visit. Eligible participants were asked to fast for at least 12 h and to refrain from caffeine, alcohol, polyphenol-containing foods/beverages, and any form of physical activity above light intensity for at least 24 h prior to each of the two experimental visits. Some polyphenol metabolites can remain in circulation after 24 h [75]; however, most metabolites are excreted within the first 24 h of intake [76]. The 2 experimental visits started in the early morning (08:00–10:30), where volunteers were invited to rest in a supine position for approximately 15 min, during which compliance with the 24 h dietary restrictions was assessed. This was followed by baseline blood pressure measurements and a pre-intervention CCA ultrasound recording to assess resting (i) anterograde and retrograde BF, (ii) anterograde and retrograde SR, and (iii) arterial diameter. Participants then sat for 2 h on a comfortable seat, beginning with the consumption of the high- or low-flavanol cocoa beverage (randomly assigned and double-blinded). While sitting, the participants were asked to keep their legs as still as possible and in a standard position (parallel and bent at approx. 90°, with both feet in a neutral position and with the plantar surface on the floor) while keeping the trunk resting on the backrest. Participants were only allowed to move their arms/hands to do light activities (e.g., computer typing). Following the 2 h sitting trial, the CCA ultrasound recording was repeated (as a post-intervention procedure). Experimental visits were performed in a quiet, darkened, and temperature-controlled laboratory (22–24 °C), as recommended in guidelines for ultrasound-related measures such as the FMD protocol [77,78]. A sitting duration of 2 h was selected because (i) this timeframe is sufficient to observe decreases in FMD following sitting [79] and (ii) it coincides with the peak bioavailability of flavanol metabolites in circulation in humans [80].

Finally, physical activity monitoring was measured using a tri-axial accelerometer and analyzed using the GENEActiv software (GENEActiv, version 3.3, ActivInsights Ltd., Kimbolton, England), as previously described [73,74].

### 2.4. Maximal Aerobic Capacity Test

The peak aerobic capacity (VO_2_peak) of young participants was assessed by a maximal incremental exercise test on a cycle ergometer (Lode Excalibur Sport, Groningen, the Netherlands). Briefly, the test commenced at 100 W and was incremented by 30 W every 2 min until at least one of the following conditions was met: (i) voluntary exhaustion, (ii) a rate of perceived exertion (RPE) score of 20 (Borg RPE scale: 6–20), or (iii) not maintaining a cadence of ≥60 rpm. During the exercise test, respiratory gas exchange measurements were taken using an automated gas analyzer (Vyntus CPX Metabolic Cart, Vyaire Medical, Mettawa, IL, USA) to obtain the rate of oxygen consumption (VO_2_).

### 2.5. High- and Low-Flavanol Interventions

Cocoa flavanol beverages were prepared as previously described [54,81]. Briefly, 12 g of high/low-flavanol cocoa powder, both of which are commercially available (Barry Callebaut AG, Zurich, Switzerland), was dissolved into 350 mL of “Buxton” still natural mineral water (nitrate: <0.1 mg/L). The beverages were prepared immediately before consumption by the volunteer. The low-flavanol cocoa powder was a fat-reduced alkalized cocoa powder containing < 6 mg of (−)-epicatechin and 5.6 mg of total flavanols per beverage. The high-flavanol cocoa powder was a fat-reduced natural cocoa powder containing 150 mg of (−)-epicatechin and 695 mg of total flavanols per beverage. This dose was informed by previous studies showing efficacy in modifying brachial FMD after flavanol intake [82,83,84]. The macro-nutrient concentrations and flavonoid composition of the interventions are reported in Table 1.

The two cocoa powders were matched for all micro- and macro-nutrients, including methylxanthines, caffeine, and theobromine. Cocoa powder concentrations for flavanol monomers, procyanidins, and methylxanthines were measured using high-performance liquid chromatography, as described in previous studies [85,86]. Total polyphenol concentrations were estimated using a Folin–Ciocalteu reagent calorimetric assay, as described previously [87]. In order to ensure double-blindness, cocoa beverages were provided in an opaque container (covered on top) with a dark-colored opaque straw and were indistinguishable in texture, aroma, and taste. Unblinding of the cocoa interventions was performed after all data analyses were completed.

### 2.6. Ultrasound Recording of the Common Carotid Artery

Common carotid artery scans were performed in accordance with ultrasound scanning guidelines [88]. The artery diameter and blood velocity of the CCA were non-invasively assessed by means of a high-resolution duplex ultrasound device (Terason uSmart 3300, Teratech Corporation, Burlington, MA, USA) with a 15–4 MHz linear array transducer (Terason 15L4 Smart Mark, Teratech Corporation, Burlington, MA, USA), using a set frequency of 5.0 MHz. Artery diameter was assessed using B-mode ultrasound imaging, while blood velocity was assessed using pulse-wave Doppler. The pulse-wave Doppler signal was corrected at an insonation angle of 60°, with the sample volume (size: 1.5 mm) positioned in the middle of the arterial lumen. The right CCA was located and scanned longitudinally in the middle area of the neck, approx. 1–2 cm proximal to the carotid bifurcation (the point at which the CCA splits into the external carotid artery and internal carotid artery). Once a satisfactory image of the artery was obtained (with defined vascular walls), the ultrasound probe was stabilized by hand, and then the artery diameter and blood velocity were continuously recorded for 2 min. The baseline diameter was calculated as the average diameter recorded during the 2 min CCA recording. Before starting the recording, the B-mode image was captured and saved in the ultrasound device in order to ensure consistency for subsequent CCA recordings. All CCA scans were performed by a trained and experienced PhD student (AD; first author) with previous experience in ultrasound-related measures [89]. The candidate’s inter-day coefficient of variation (CV) for CCA BF and SR was 9.0% and 10.5%, respectively, and 1.7% for the CCA diameter.

Measurements of the artery diameter and blood velocity were analyzed offline using an automated edge detection software (Cardiovascular Suite, version 3.4.0, Quipu S.r.l., Pisa, Italy). Video recordings were analyzed by the same researcher (AD) who performed the CCA recordings. The time-averaged maximum velocity, as calculated by the software, was adjusted to reflect the time-averaged mean velocity as reported in previous research [90,91], using the following formula:

$$\mathrm{Time}\;\mathrm{Averaged}\;\mathrm{Mean}\;\mathrm{Velocity}=\frac{\mathrm{Time}\;\mathrm{Averaged}\;\mathrm{Maximum}\;\mathrm{Velocity}}{2}$$

The resulting blood velocity was utilized to calculate the BF using the following formula:

$$\mathrm{Blood}\;\mathrm{Flow}=\left[\left.\mathrm{Blood}\;\mathrm{Velocity}\;\times \;\pi {\left.\left(\frac{\mathrm{Baseline}\;\mathrm{Diameter}}{2}\right.\right)}^{2\;}\right]\right.\times \;60$$

Although the shear rate does not incorporate the inherent viscous properties of blood [92], the shear rate provides an appropriate surrogate measure of shear stress [93] and was calculated as described below:

$$\mathrm{Shear}\;\mathrm{Rate}=\frac{4\;\times \;\mathrm{Baseline}\;\mathrm{Blood}\;\mathrm{Velocity}}{\mathrm{Baseline}\;\mathrm{Diameter}}$$

### 2.7. Blood Pressure and Heart Rate

Resting brachial systolic and diastolic blood pressure and heart rate were measured in the left upper arm in the supine position (after >10 min of resting in the supine position) using an automatic blood pressure monitor (Omron M3 [HEM-7131-E], OMRON HEALTHCARE Co., Ltd., Kyoto, Japan) (data presented in [73,74]).

### 2.8. Statistical Analysis

All statistical analyses were performed using the statistical software IBM SPSS Statistics for Windows, version 29 (IBM Corp., Armonk, NY, USA). One-way ANOVA was performed to determine differences in participants’ baseline characteristics between young (high fit; low fit) and older groups. Analysis of the CCA-related outcome measures was performed separately for young and older adults, as these studies were conducted independently. For the young group, three-way repeated measures ANOVA with sitting time (0; 2 h) and dietary intervention (low flavanol; high flavanol) as within-subject variables and fitness (high fit; low fit) as a between-subjects variable was performed to analyze changes in CCA parameters (i.e., arterial diameter, BF, and SR). For the older group, two-way repeated measures ANOVA with sitting time (0; 2 h) and dietary intervention (low flavanol; high flavanol) as within-subject variables was performed to analyze changes in CCA parameters (i.e., arterial diameter, BF, and SR). When statistical significance was found for interaction effects, Bonferroni post hoc analysis was performed. The data in the tables and figures are presented as the mean ± SD. A *p*-value of less than 0.05 was considered statistically significant.

## 3. Results

### 3.1. Study Participants

Participants’ anthropometric and physiological characteristics are summarized in Table 2. Participants were young healthy males—divided into two fitness groups (high fit [HF]: age, 22.2 ± 2.9 yr. [*N* = 20]; low fit [LF]: age, 23.2 ± 4.1 yr. [*N* = 20])—and healthy older adults (age, 72.4 ± 5.0 yr. [males, *N* = 7; females, *N* = 13; total, *N* = 20]), all of whom had a healthy body mass index (BMI) and heart rate. The high-fit group had a higher VO_2_ peak than the low-fit group (56.4 ± 5.3 vs. 34.8 ± 4.6 mL∙kg^−1^∙min^−1^; *p* < 0.001), as intended. BMIs were similar between young LF and older individuals, whilst BMIs were lower for young HF individuals when compared to young LF individuals (*p* < 0.01). In terms of vascular parameters, the older group had lower CCA anterograde SR than the young HF group, and higher CCA anterograde BF than the young LF group (all *p* < 0.05, see Table 2). Young LF and older adults had similar systolic BP, whilst the young HF group had lower systolic BP in comparison with the older group (*p* < 0.01). Young HF had lower diastolic BP compared to both young LF (*p* < 0.01) and older groups (*p* < 0.001); young LF individuals had lower diastolic BP than the older group (*p* < 0.01). Heart rates were similar between the young LF and older groups, whilst young HF individuals had a lower HR in comparison with both young LF (*p* < 0.01) and older groups (*p* < 0.01). In the older group, medication use is reflective of the wider older population in the UK, specifically medication for hypertension (e.g., statins; *N* = 4), hypothyroidism (e.g., levothyroxine; *N* = 3), dyspepsia (e.g., omeprazole; *N* = 1), and osteoporosis (e.g., alendronic acid; *N* = 1).

### 3.2. Habitual Physical Activity

Participants’ daily sedentary time and physical activity levels are reported in Table 3, which combines and compares the data reported elsewhere [73,74]. Both young and older participants reported similar amounts of time spent on sedentary and moderate activities. Older participants reported more light activity time and corresponding estimated MET∙min than both young HF (*p* < 0.05) and LF groups (*p* < 0.01). Older and young LF groups reported similar vigorous activity times and estimated MET∙min values, whilst young HF participants reported more vigorous activity time and higher estimated MET∙min values than both young LF (*p* < 0.001) and older individuals (*p* < 0.001). All three groups reported a similar step count.

### 3.3. Common Carotid Artery (CCA) Vascular Measures

A summary of the resting vascular parameters (arterial diameter, BF, and SR) assessed in the CCA before and after sitting following either a low- or high-flavanol intervention is presented for young and older individuals in Table 4 and Table 5, respectively.

**Arterial diameter:** Sitting had no significant impact on CCA diameter in both young (Table 3) and older groups (Table 4). In the young group only, CCA diameter is influenced by an interaction between fitness and intervention (flavanol × fitness: *p* = 0.017, η_p_^2^ = 0.142), with further post hoc analysis indicating significant differences between flavanol interventions only within the high-fit group (*p* = 0.009).

**Figure 2:**
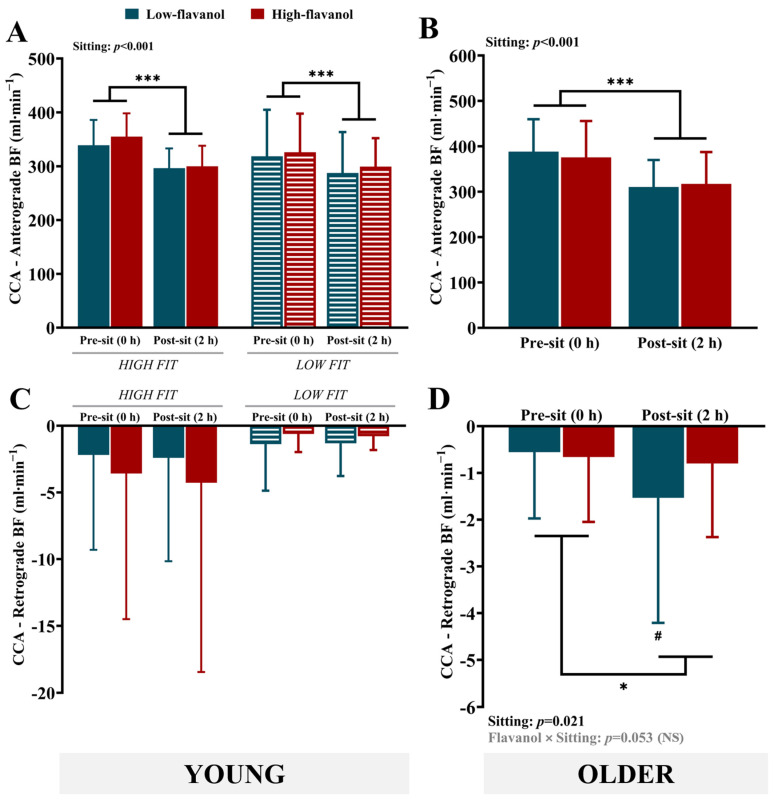
Baseline anterograde and retrograde blood flow of the common carotid artery in young (high and low fit) (**A**,**C**) and older participants (**B**,**D**), before (0 h) and after 2 h of sitting, following either a low- or high-flavanol intervention. Data are presented as mean ± SD. * Denotes a significant difference (*p* < 0.05) between pre- and post-sitting. *** Denotes a significant difference (*p* < 0.001) between pre- and post-sitting. # Denotes a significant difference (*p* < 0.05) between pre- and post-sitting within the low-flavanol intervention. BF, blood flow; CCA, common carotid artery. The data were derived from secondary analyses of two RCTs that were not specifically powered to detect changes in CCA blood flow.

**Blood flow:** Sitting resulted in a significant decline in CCA anterograde BF in both younger groups (*p* < 0.001, η_p_^2^ = 0.496) (Figure 2A), as well as in the older group (*p* < 0.001, η_p_^2^ = 0.674) (Figure 2B), with no effect of the flavanol intervention. In regard to CCA retrograde BF (Figure 2C,D), only the older group displayed a significantly greater negative flow following the sitting protocol (*p* = 0.021, η_p_^2^ = 0.251) (Figure 2D). The older group also displayed a non-significant trend for the flavanol × sitting interaction (*p* = 0.053, η_p_^2^ = 0.183), with further post hoc analysis indicating that there was an increase in negative flow with sitting only after the low-flavanol intervention (*p* = 0.028), whilst after the high-flavanol intervention, there was no significant increase in negative flow (*p* = 0.221). No significant differences between the high- and low-flavanol interventions were detected post-sitting for retrograde BF (*p* = 0.201, η_p_^2^ = 0.084).

**Figure 3:**
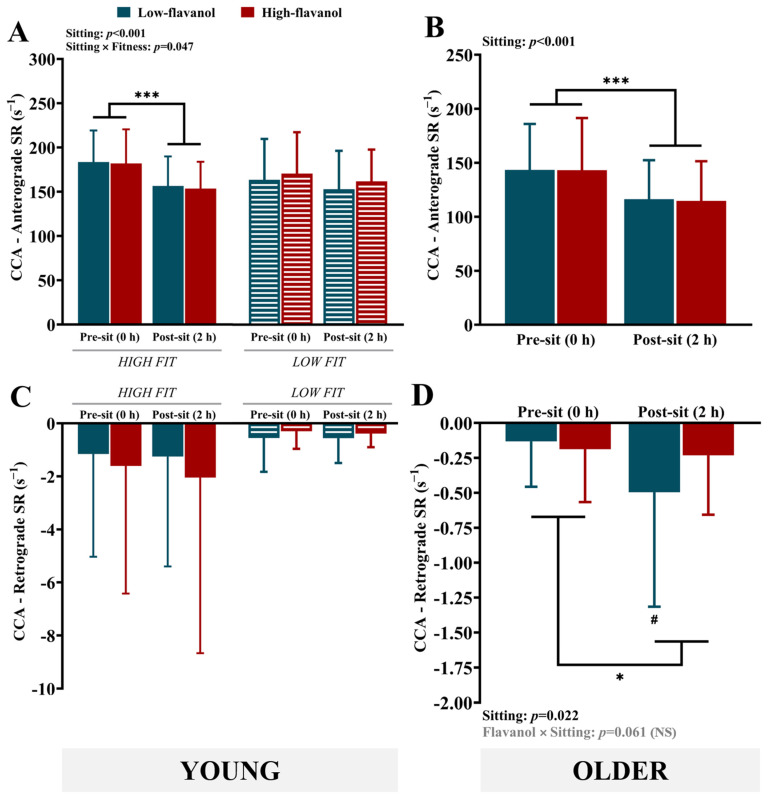
Baseline anterograde and retrograde shear rates of the common carotid artery in young (high and low fit) (**A**,**C**) and older participants (**B**,**D**) before (0 h) and after 2 h of sitting, following either a low- or high-flavanol intervention. Data are presented as the mean ± SD. * Denotes a significant difference (*p* < 0.05) between pre- and post-sitting. *** Denotes a significant difference (*p* < 0.001) between pre- and post-sitting. # Denotes a significant difference (*p* < 0.05) between pre- and post-sitting within the low-flavanol intervention. CCA, common carotid artery; SR, shear rate. The data were derived from secondary analyses of two RCTs that were not specifically powered to detect changes in CCA shear rate.

**Shear rate:** Sitting resulted in a significant reduction in anterograde SR in the high-fit group (*p* < 0.001) but not in the low-fit group (Figure 3A). Similarly, sitting-induced declines in anterograde SR were also observed in the older group (*p* < 0.001, η_p_^2^ = 0.737) (Figure 3B). Flavanol interventions had no impact on anterograde SR. Similar to what was observed for BF, sitting had no effect on retrograde SR in either of the young groups (Figure 3C) but resulted in an increase in the older group (*p* = 0.022, η_p_^2^ = 0.246) (Figure 3D). Additionally, there was a non-significant trend for the flavanol × sitting interaction (*p* = 0.061, η_p_^2^ = 0.172), consistent with what was observed for BF, with sitting inducing an increase in negative SR following the low-flavanol intervention (*p* = 0.033) but not the high-flavanol intervention (*p* = 0.243). No significant differences between the high- and low-flavanol interventions were detected post-sitting for retrograde SR (*p* = 0.169, η_p_^2^ = 0.097).

## 4. Discussion

The main findings were that 2 h of uninterrupted sitting induced reductions in anterograde BF in all groups and anterograde SR only in young high-fit and older participants. Furthermore, only the older group experienced elevations in both retrograde BF and SR following sitting, with a potential for the high-flavanol intervention to prevent such increases. Although this effect did not reach the statistical significance threshold (interaction effect for BF: *p* = 0.053; SR: *p* = 0.061), post hoc analyses show that only the low-flavanol cocoa led to significant increases in retrograde BF and SR during sitting. No changes in arterial diameter were detected due to sitting or flavanol interventions.

### 4.1. Impact of Sitting on Carotid Blood Flow/Shear Rate: Role of Fitness and/or Aging

In the present study, both young and older individuals experienced sitting-induced reductions in anterograde BF. No significant differences were detected between the young fitness groups, suggesting that higher cardiorespiratory fitness does not prevent the effects of sitting on anterograde cerebral BF. Relative reductions in anterograde BF and SR seem to be more pronounced in the older group (BF: −17.8%, −68.1 mL∙min^−1^; SR: −19.4%, −27.8 s^−1^) compared to the young high-fit (BF: −14.1%, −48.8 mL∙min^−1^; SR: −15.2%, −27.8 s^−1^) and low-fit groups (BF: −9.0%, −29.0 mL∙min^−1^; SR: −5.9%, −9.8 s^−1^). Importantly, only older individuals experienced additional significant increases in retrograde BF and SR (BF: 150.0%, 0.9 mL∙min^−1^; SR: 400.0%, 0.4 s^−1^), whilst young high-fit (BF: 9.1%, 0.2 mL∙min^−1^; SR: 8.3%, 0.1 s^−1^) and low-fit participants (BF: −7.1%, −0.1 mL∙min^−1^; SR: 0.0%, 0.0 s^−1^) did not. This is perhaps to be expected, as older adults tend to display signs of deterioration in vascular structure and function, particularly increases in carotid artery intima-media thickness (IMT) [94], which is a biomarker of subclinical atherosclerosis [95,96]. Other negative hallmarks are carotid plaque accumulation [97], carotid artery stenosis [98], and lower cerebrovascular reactivity [99], as well as reduced resting cerebral perfusion [100,101,102], all of which are early signs of cerebrovascular impairments [103,104]. Importantly, there were no differences in baseline (prior to sitting) resting retrograde BF and SR between young and older groups, indicating that the differences detected after sitting are not just a reflection of differences at baseline but emerge as a consequence of prolonged sitting. Therefore, this observation indicates that older individuals may be more vulnerable to the acute effects of sitting, with potentially negative consequences for arterial endothelial function. For example, acute reductions in anterograde SR are known to have a negative impact on endothelial function (assessed using FMD) in the upper-limb brachial artery [67]. Shear rate is a surrogate measure of shear stress [93] and a major determinant of vascular health: low shear stress is considered atherogenic (promotes atherosclerosis development) [105]. In the CCA specifically, lower wall shear stress has been found to be associated with higher plasma viscosity and intima-media thickness, which can have implications for the long-term risk of carotid plaque development [106]. Similarly, Thijssen et al. [66] have demonstrated that acute exposure to elevated retrograde SR induces dose-dependent decreases in brachial artery FMD [66]. Notwithstanding that only resting SR measures were assessed in the present study, the increases in retrograde SR and decreases in anterograde SR observed may be indicative of a sitting-induced decline in carotid endothelial function. The observed decline in anterograde SR in both age groups following sitting suggests a possible pathway through which sitting could impair vascular health, with endothelial NO production being a likely contributor, given that laminar shear stress stimulates the release of NO from endothelial cells [107].

Only two previous studies in older adults have looked at the impact of sitting on the brain’s cerebrovasculature, reporting contrasting results. Wheeler et al. [31] report sitting-induced reductions in MCA blood velocity, whilst Maasakkers et al. [39] report no changes in MCA blood velocity. This could be attributed to some differences between the studies, including the duration of the sitting protocol (8 h vs. 3 h, respectively) and sample size (*N* = 12 vs. *N* = 22, respectively). Interestingly, an increase in cerebrovascular resistance was also reported in the study of Maasakkers et al. [39], which is in alignment with the observations of the current study, given that increases in retrograde SR/BF have been associated with higher arterial resistance [108]. Whether the reported changes in cerebral vascular physiology post-sitting have acute consequences for cognition in older adults is currently unclear and should be the focus of future research [39,109,110,111].

It is well-established that higher fitness has positive effects on vascular health, including cerebrovascular health [58,112]. Findings from the present study suggest that levels of fitness in younger adults seem to have little impact on cerebral BF during sitting, with both high- and low-fit groups experiencing similar declines in anterograde BF. Previous findings in young adults generally report sitting-induced reductions in MCA blood velocity [30,38], ICA BF [32,34,113], and CCA BF [33]. Contrary to one previous study suggesting CCA vasoconstriction during sitting [37], in the present study, there was no change in the arterial diameter as a result of the sitting protocol.

Finally, previous work has reported a significant reduction in cerebral oxygenation during cognitive performance post-sitting (the Flanker Task, which assesses selective attention and inhibitory function) among physically active young adults, but not among those who were physically inactive [114]. In line with this, in the current study, only the high-fit group displayed significant reductions in anterograde SR in the CCA, with no changes detected in the low-fit group. It is challenging to interpret this observation as it suggests that the high-fit group may be more susceptible to sitting. However, declines in anterograde BF during sitting were similar and significant in both fitness groups. Future studies should address this question by using a more comprehensive set of outcome measures, including resting and functional measures (e.g., CO_2_ reactivity), as well as cognitive assessments, to compare the effects of fitness on cerebrovascular function during sitting.

### 4.2. Impact of Flavanols on Carotid Blood Flow/Shear Rate During Sitting

Findings from the present study suggest a possible beneficial effect of cocoa flavanol intake on alleviating the impact of sitting on increases in retrograde BF and SR in older individuals but not in young adults. This was indicated by a near-significant interaction effect for both retrograde BF (*p* = 0.053) and SR (*p* = 0.061), with post hoc tests detecting that only the low-flavanol condition experienced significant elevations post-sitting. As these are secondary outcome measures of the original RCTs, this study was not *a priori* powered to detect differences between treatments. Post-study power calculations indicated that *N* = 22 and *N* = 25 would be sufficient to detect potential differences between flavanol treatments for retrograde SR and BF post-sitting in older adults, respectively (power = 0.95; *p* = 0.05, *f* = 0.30–0.33). This confirms that our study was slightly underpowered to detect these effects but provides some useful information regarding sample sizes for future studies.

This is the first study to suggest that flavanols may have the potential to modulate cerebral retrograde BF and SR. Although we are unable to confirm this in the present study, reductions in retrograde BF and SR, which are inversely related to NO production [115], are known to result in improvements in endothelial function (as measured by FMD) [116]. Previous work in young healthy adults (no sitting) reports that the intake of cocoa flavanols does not modulate resting anterograde or retrograde carotid BF [81], which is in agreement with the current data in the young group. However, this has never been investigated in older adults.

There is some evidence to suggest that the benefits from flavonoid/flavanol intake are more pronounced in older adults than in young adults [117,118]. Thus, it is possible that these positive effects only emerged in the older group because sitting induced an endothelial-dependent functional deficit in the vasculature of the older adults. It is currently unclear whether prolonged sitting results in cerebral endothelial dysfunction, but there is a wealth of evidence indicating that it does in the peripheral vasculature [119]. In this regard, one of the main mechanisms underlying the effects of flavanol metabolites on the arterial endothelium is thought to be the increased production and bioavailability of NO [120,121,122] through NO-induced vasodilation. Recent evidence highlights that cocoa flavanols can modulate cerebral oxygenation during NO-dependent hypercapnia [54], indicating that these mechanisms might also be at play in the cerebral vasculature. Nevertheless, we cannot confirm that the observed effect is attributable to NO-mediated mechanisms, as our study did not include measurements of this variable. Previous studies on the acute effects of cocoa flavanols on the brain vasculature indicate that beneficial effects may be detected in both young and older individuals, yet studies report generally contradictory results, especially when effects are assessed in the resting state [53,55,123] or in response to physiological/cognitive challenges [52,124,125,126]. As such, cerebral functional measures targeting NO-related mechanisms should be used in future sitting studies [54]. For example, ICA vasodilation in response to hypercapnia has been shown to be NO-dependent, and it is considered a good surrogate marker of cerebral endothelial function (“ICA FMD”) [127,128,129]. By using a combination of resting BF/SR in the ICA and “ICA FMD” in the context of sitting, future studies will be able to determine whether flavanols’ modulation of retrograde SR/BF in older adults during sitting results in improvements in cerebral endothelial function.

Peripheral endothelial function is known to be impaired after menopause [130], with some evidence also suggesting impairments in cerebral blood flow [131] due to declines in estrogen levels. As such, the intake of flavonoids in postmenopausal women may be particularly beneficial in this population [126,132,133,134,135]. In the present study, we were not adequately powered to detect sex differences, so future studies should investigate whether flavanol-rich dietary options are more effective in protecting cerebral blood flow during prolonged sitting in postmenopausal women compared to age-matched men.

Finally, previous studies have indicated that older populations with a lower flavonoid intake may benefit more from the addition of flavonoids to their diet [136]. Our studies suggest age differences in habitual flavonoid intake (reported elsewhere [73,74]), with older adults (863.4 mg/day) consuming more than young adults (331.2 mg/day), as previously described for the UK population [137,138]. A similar pattern is observed for flavanol intake (older: 382.4 mg/day; young: 172.9 mg/day), with tea and fruit (grapes, berries, and apples) being the most substantial contributors within the diet. Our preliminary data in older adults suggest that benefits may still be present when habitual flavonoid intake is higher. Alternatively, the effect of aging is more prevalent in driving the beneficial effects of flavonoid consumption in the context of sitting. More RCTs are needed to formally address this question.

## 5. Limitations

The current study presents some limitations. Firstly, the CCA vascular measures presented in this study were secondary outcome measures of two RCTs [73,74], which were powered based on peripheral endothelial function (FMD). As such, future studies should include a sample size of at least *N* = 25, according to power calculations estimated based on current differences in retrograde BF and SR between interventions post-sitting. Secondly, comparisons of the effects of flavanols between the young and older groups were not directly addressed, given that these were two independent RCTs. Thirdly, the impact of physical fitness in older adults was not addressed due to safety-related constraints associated with performing a VO_2_max test in this population. Therefore, upcoming research should explore whether cardiorespiratory fitness mediates the impact of flavanols on cerebral blood flow during sitting. Fourthly, only male adults were included within the young cohort; the exclusion of females from the study cohort restricts our ability to assess potential sex-related differences in carotid vascular measures in response to sitting and flavanol intake. Consequently, our results may not be directly applicable to female populations, highlighting a need for further research to explore these effects in diverse demographic groups. Future work is needed to address the efficacy of flavanols during sitting in females. Furthermore, an equal distribution of participants across sexes in the older group would have enhanced the generalizability of our findings in this population. Finally, although the CCA is a major vessel that supplies the brain, it is acknowledged that approx. 70% of the blood that runs through it reaches the ICA [139], which serves as the primary artery supplying the brain [140]. This means that the remaining ~30% is directed to the external carotid artery, which supplies extracranial structures of the head and does not perfuse the brain [141]. Thus, measuring other arteries that perfuse the brain (e.g., the ICA and vertebral artery) in addition to the CCA would further improve understanding of cerebral hemodynamics in response to prolonged sitting and flavanol interventions. Most importantly, future studies should combine resting measures with functional measures targeting NO in the brain, as well as assessments of cognitive performance post-sitting.

## 6. Conclusions

To the best of our knowledge, the current study is the first to investigate the acute effects of dietary plant-derived flavanols on the cerebral vasculature in response to prolonged sitting in young (high/low cardiorespiratory fitness) and older adults. The findings suggest that flavanols may have the potential to attenuate the detrimental sitting-induced augmentations of retrograde BF and SR in older adults, although larger well-powered RCTs are required to confirm this. Furthermore, both young and older adults experienced declines in anterograde BF after sitting, but only older adults experienced increases in retrograde BF. Our preliminary data highlight that food choices during periods of sitting might have the potential to minimize the impact of inactivity on resting cerebral BF in older adults. Additionally, to further enhance our understanding of flavonoid intake during sitting and its implications for neurological health, it is crucial that future studies adopt chronic interventions and assess cognitive outcomes, thereby providing a more comprehensive picture of the cerebrovascular benefits of dietary flavonoids in the context of sedentary behavior.

### Practical Applications

Given the high prevalence of sedentary time, especially in older populations, and its association with accelerated declines in brain function and cognition, it is crucial to develop efficacious strategies that can be used alongside physical activity initiatives to attenuate the adverse effects of sitting on the brain. In situations where prolonged sitting is unavoidable, the consumption of polyphenol-rich foods such as green tea, black tea, unprocessed cocoa, apples, and berries may help alleviate the negative impact on cerebrovascular health. The flavanol dose used in our study was relatively high but could be achieved through the intake of two cups of green, black tea, or unprocessed cocoa or a combination of apples, berries, and green/black tea [142]. Future studies should establish the minimum doses necessary to confer beneficial effects in the context of sitting. Furthermore, different types of flavonoids might be explored in the future to prolong the cardiovascular benefits beyond 2 h of sitting, for example, by combining anthocyanin-rich berries or flavanol-rich green tea with flavanone-rich citrus fruits, which can improve endothelial function across different time frames [143,144]. Implementing dietary interventions rich in polyphenols could offer a practical public health strategy to mitigate the cardiovascular risks associated with sedentary behavior. In particular, cocoa flavanols have been demonstrated to reduce the risk of cardiovascular events [136]. Therefore, strategically incorporating these compounds during sedentary periods, when the cerebrovasculature may be more vulnerable, has the potential to enhance overall health benefits over time.

## Figures and Tables

**Table 1:** Nutritional composition of the high- and low-flavanol cocoa powders (12 g per individual dose).

	Low Flavanol	High Flavanol
Total polyphenols *	260.0	1246.8
Total flavanols (mg)	5.6	695.0
Procyanidins (dimers-decamers; mg)	ND	459.6
(−)-Epicatechin (mg)	<6	150.0
(−) and (+)-Catechin (mg)	<6	85.4
Theobromine (mg)	278.4	262.8
Caffeine (mg)	22.2	27.6
Fat (g)	1.3	1.7
Carbohydrates (g)	1.2	2.7
Protein (g)	2.7	2.7
Fiber (g)	4.0	1.8
Energy (kcal)	36.6	41.4

* Concentrations are expressed as mg of gallic acid equivalents per 12 g of cocoa powder.

**Table 2:** Participants’ baseline characteristics.

	Young		Older
	High Fit	Low Fit	
*N*	20	20	20
**Anthropometric and Fitness**			
Age (yr.)	22.2 ± 2.9 $	23.2 ± 4.1 $	72.4 ± 5.0
Height (m)	1.76 ± 0.06 ¢	1.79 ± 0.09 $	1.67 ± 0.10
Weight (kg)	70.4 ± 8.8 **	83.5 ± 11.9 ¢	68.7 ± 15.6
BMI (kg∙m^−2^)	22.7 ± 2.5 **	26.0 ± 2.3	24.4 ± 3.6
VO_2_peak (mL∙kg^−1^∙min^−1^)	56.4 ± 5.3 ***	34.8 ± 4.6	N/A
**Common Carotid Artery**			
Artery diameter (mm)	6.9 ± 0.3 $	6.9 ± 0.5 $	7.8 ± 0.8
Anterograde blood flow (mL∙min^−1^)	347.0 ± 41.3	322.2 ± 73.8 ¥	382.0 ± 66.9
Retrograde blood flow (mL∙min^−1^)	−2.9 ± 9.0	−1.0 ± 2.3	−0.6 ± 1.3
Anterograde shear rate (s^−1^)	182.8 ± 34.3 ¢	167.0 ± 42.8	143.3 ± 43.9
Retrograde shear rate (s^−1^)	−1.4 ± 4.3	−0.4 ± 0.9	−0.2 ± 0.3
**Blood Pressure and Heart Rate**			
Systolic BP (mm Hg)	117.9 ± 9.1 ¢	121.3 ± 8.5	128.4 ± 12.6
Diastolic BP (mm Hg)	63.2 ± 5.4 **$	68.8 ± 4.5 ¢	74.5 ± 6.7
Heart rate (bpm)	50.9 ± 7.4 **¢	63.1 ± 9.6	61.5 ± 11.5

Data are presented as the mean ± SD. ** Denotes a significant difference (*p* < 0.01) between the two fitness groups within the same parameter. *** Denotes a significant difference (*p* < 0.001) between the two fitness groups within the same parameter. ¥ Denotes a significant difference (*p* < 0.05) between young and older individuals within the same parameter. ¢ Denotes a significant difference (*p* < 0.01) between young and older individuals within the same parameter. $ Denotes a significant difference (*p* < 0.001) between young and older individuals within the same parameter. BMI, body mass index; BP, blood pressure; VO_2_, rate of oxygen consumption.

**Table 3:** Participants’ daily sedentary time and physical activity.

	Young		Older
Daily Physical Activity Pattern	High Fit	Low Fit	
Sedentary activity time (h)	10.4 ± 1	10 ± 2.2	9.8 ± 1.5
Light activity time (h)	1.4 ± 0.4 ¥	1.2 ± 0.4 ¢	1.8 ± 0.6
Moderate activity time (h)	2.8 ± 0.5	2.6 ± 0.8	2.5 ± 1.1
Vigorous activity time (h)	0.6 ± 0.4 ***$	0.1 ± 0.1	0.0 ± 0.1
Sedentary activity estimated MET∙min (mL∙kg^−1^∙min^−1^)	765.2 ± 80.3	716.3 ± 158.4	747.1 ± 138.7
Light activity estimated MET∙min (mL∙kg^−1^∙min^−1^)	200.5 ± 52.7 ¥	176.5 ± 63.4 ¢	263.6 ± 95.3
Moderate activity estimated MET∙min (mL∙kg^−1^∙min^−1^)	685.1 ± 146.5	622.5 ± 205.2	533.7 ± 243.6
Vigorous activity estimated MET∙min (mL∙kg^−1^∙min^−1^)	336.0 ± 286.9 ***$	70.0 ± 75.2	25.2 ± 35.3
Step count	12,421.7 ± 2333.8	9896.5 ± 3663.6	10,651.8 ± 3883.6

Data are presented as the mean ± SD. *** Denotes a significant difference (*p* < 0.001) between the two fitness groups within the same parameter. ¥ Denotes a significant difference (*p* < 0.05) between young and older individuals within the same parameter. ¢ Denotes a significant difference (*p* < 0.01) between young and older individuals within the same parameter. $ Denotes a significant difference (*p* < 0.001) between young and older individuals within the same parameter. MET, metabolic equivalent of task.

**Table 4:** Vascular parameters of the common carotid artery in young (high- and low-fit) participants, before and after the 2 h sitting protocol following either a low- or high-flavanol intervention.

	High Fit				Low Fit				
	Low Flavanol		High Flavanol		Low Flavanol		High Flavanol		
CCA	Pre	Post	Pre	Post	Pre	Post	Pre	Post	Effect
Artery diameter (mm)	6.8 ± 0.3 **	6.9 ± 0.3 **	7.0 ± 0.3	7.0 ± 0.4	6.9 ± 0.6	6.9 ± 0.6	6.9 ± 0.5	6.8 ± 0.6	Flavanol × Fitness (*p* = 0.017)
Anterograde BF (mL∙min^−1^)	339.0 ± 47.0	296.4 ± 36.6	354.9 ± 43.4	299.9 ± 38.0	318.5 ± 86.4	287.3 ± 76.1	326.0 ± 71.7	299.2 ± 52.9	Sitting (*p* < 0.001)
Retrograde BF (mL∙min^−1^)	−2.2 ± 7.1	−2.4 ± 7.7	−3.6 ± 10.9	−4.3 ± 14.2	−1.4 ± 3.5	−1.3 ± 2.4	−0.6 ± 1.4	−0.8 ± 1.0	NS
Anterograde SR (s^−1^)	183.6 ± 35.6 ***	156.5 ± 33.4	182.0 ± 38.5 ***	153.6 ± 30.3	163.5 ± 46.0	152.8 ± 43.4	170.6 ± 46.6	161.7 ± 35.8	Sitting (*p* < 0.001); Sitting × Fitness (*p* = 0.047)
Retrograde SR (s^−1^)	−1.2 ± 3.9	−1.3 ± 4.1	−1.6 ± 4.8	−2.0 ± 6.6	−0.6 ± 1.3	−0.6 ± 0.9	−0.3 ± 0.7	−0.4 ± 0.5	NS

Data are presented as the mean ± SD. ** Denotes a significant difference (*p* < 0.01) between flavanols within the same fitness group. *** Denotes a significant difference (*p* < 0.001) between pre- and post-sitting within the same fitness group. BF, blood flow; CCA, common carotid artery; SR, shear rate. The data were derived from secondary analyses of two RCTs that were not specifically powered to detect changes in CCA blood flow and shear rate.

**Table 5:** Vascular parameters of the common carotid artery in older participants before and after the 2 h sitting protocol following either a low- or high-flavanol intervention.

	Low Flavanol		High Flavanol		
CCA	Pre	Post	Pre	Post	Effect
Artery diameter (mm)	7.8 ± 0.8	7.8 ± 0.8	7.8 ± 0.8	7.9 ± 0.8	NS
Anterograde BF (mL∙min^−1^)	388.4 ± 71.3	310.6 ± 59.4	375.6 ± 80.2	317.2 ± 70.2	Sitting (*p* < 0.001)
Retrograde BF (mL∙min^−1^)	−0.6 ± 1.4	−1.5 ± 2.7	−0.7 ± 1.4	−0.8 ± 1.6	Sitting (*p* = 0.021); Flavanol × Sitting (*p* = 0.053) (NS)
Anterograde SR (s^−1^)	143.5 ± 42.5	116.3 ± 36.1	143.1 ± 48.3	114.7 ± 36.7	Sitting (*p* < 0.001)
Retrograde SR (s^−1^)	−0.1 ± 0.3	−0.5 ± 0.8	−0.2 ± 0.4	−0.2 ± 0.4	Sitting (*p* = 0.022); Flavanol × Sitting (*p* = 0.061) (NS)

Data are presented as the mean ± SD. BF, blood flow; CCA, common carotid artery; SR, shear rate. The data were derived from secondary analyses of two RCTs that were not specifically powered to detect changes in CCA blood flow and shear rate.

## Data Availability

The data that support the findings of this study are available from the corresponding author (CR) upon reasonable request.
